# Metabolomics and the pig model reveal aberrant cardiac energy metabolism in metabolic syndrome

**DOI:** 10.1038/s41598-020-60387-7

**Published:** 2020-02-26

**Authors:** Maryam Karimi, Victoria Petkova, John M. Asara, Michael J. Griffin, Frank W. Sellke, Alan R. Bishop, Boian S. Alexandrov, Anny Usheva

**Affiliations:** 1 Division of Cardiothoracic Surgery, Department of Surgery, The Warren Alpert Medical School, Brown University, Providence, RI 02903 United States; 2 Beth Israel Deaconess Medical Center, Harvard Medical School, Boston, MA 02115 United States; 3 Sam Houston State University, College of Osteopathic Medicine, Huntsville, TX 77320 United States; 4 Los Alamos National Laboratory, Los Alamos, NM 87545 United States

**Keywords:** Glycobiology, Metabolic syndrome

## Abstract

Although metabolic syndrome (MS) is a significant risk of cardiovascular disease (CVD), the cardiac response (MR) to MS remains unclear due to traditional MS models’ narrow scope around a limited number of cell-cycle regulation biomarkers and drawbacks of limited human tissue samples. To date, we developed the most comprehensive platform studying MR to MS in a pig model tightly related to human MS criteria. By incorporating comparative metabolomic, transcriptomic, functional analyses, and unsupervised machine learning (UML), we can discover unknown metabolic pathways connections and links on numerous biomarkers across the MS-associated issues in the heart. For the first time, we show severely diminished availability of glycolytic and citric acid cycle (CAC) pathways metabolites, altered expression, GlcNAcylation, and activity of involved enzymes. A notable exception, however, is the excessive succinate accumulation despite reduced succinate dehydrogenase complex iron-sulfur subunit b (SDHB) expression and decreased content of precursor metabolites. Finally, the expression of metabolites and enzymes from the GABA-glutamate, GABA-putrescine, and the glyoxylate pathways significantly increase, suggesting an alternative cardiac means to replenish succinate and malate in MS. Our platform discovers potential therapeutic targets for MS-associated CVD within pathways that were previously unknown to corelate with the disease.

## Introduction

One of the most concerning implications of the rapidly expanding MS is the increased risk of CVD^1^. Many processes underlying the cardiac response to MS, however, are not yet clarified. There is little data on cross-talk between metabolites alterations and cardiac functionality. MS is a state of simultaneously appearing at least three of the medical conditions elevated triglycerides, low-density lipoprotein (***LDL***), blood pressure, hyperglycemia, and obesity^1,2^. Each of these factors independently and synergistically increase the risk of developing CVD^1^. Because of the pleiotropic nature of CVD and MS, it is unlikely that single metabolites or metabolic pathways underlie the development of CVD in MS. To this end, understanding the global metabolic state of the MS heart, using high throughput metabolomics, transcriptomics, and proteomics, may provide novel insights.

Although progress has been made in characterizing some signaling mechanisms and proteins involved in the cardiac response to MS, metabolic processes implicated in the altered cardiac energy demands may contribute to the CVD response in MS as well^3,4^. In a recent metabolomics study, some amino acids and derivatives were shown to be altered in blood samples from adult humans with MS^1,2^.

Given the unique metabolic properties of the heart, analyses of metabolite levels in the blood and other body fluids, however, likely do not accurately reflect changes in this tissue. Furthermore, limited human tissue samples availability and metabolic pathway discrepancies between human and small animal species complicate the clarification of the cardiac response to MS, necessitating different approaches in the characterization of MS^5^.

Therefore, the present study aimed to use a pig-based model that recapitulates the human MS criteria when fed a high-fat high-calorie diet^6,7^. Control lean diet (LD) and MS pig heart were harvested and used to generate profiles of 283 polar metabolites by applying liquid chromatography-tandem mass spectrometry (LC/MS-MS) and thin-layer chromatography (TLC) as well as to generate gene expression profile by whole transcriptome shotgun sequencing (RNA-Seq). We then apply unsupervised machine learning (UML) to differentiate metabolite profiles from MS and LD pigs together with transcriptomics, enzymatic activities, and physiological data. We discovered that the heart adopts a strategy to defend against MS by severely altering glycolysis, the availability of several Krebs’s cycle (Citric Acid Cycle, CAC) intermediates, and changes in CAC related enzyme expression, GlcNAcylation, and activity. Our studies suggest that increased activity of the gamma-aminobutyric acid (GABA) and glyoxylate pathways replenish CAC intermediates in MS. These observations point towards a cardiac obligation in MS to modulate the expression of enzymes involved in that GABA-glutamate, GABA-putrescine, and glyoxylate cycles that could explain the accumulation of succinate and malate in MS. Overall, with our platform, we discovered unknown pathway connections and correlations on numerous biomarkers across all five MS-associated factors leading to a deeper and broader insight into CVD pathogenesis. We plan to continue to leverage our pig-based platform’s capabilities to discover and investigate potential therapeutic targets to MS-associated CVD within pathways that were previously unknown to corelate with the disease.

## RESULTS

### A high-fat/high-calorie diet leads to MS-related alterations in our pig model

**Figure 1:**
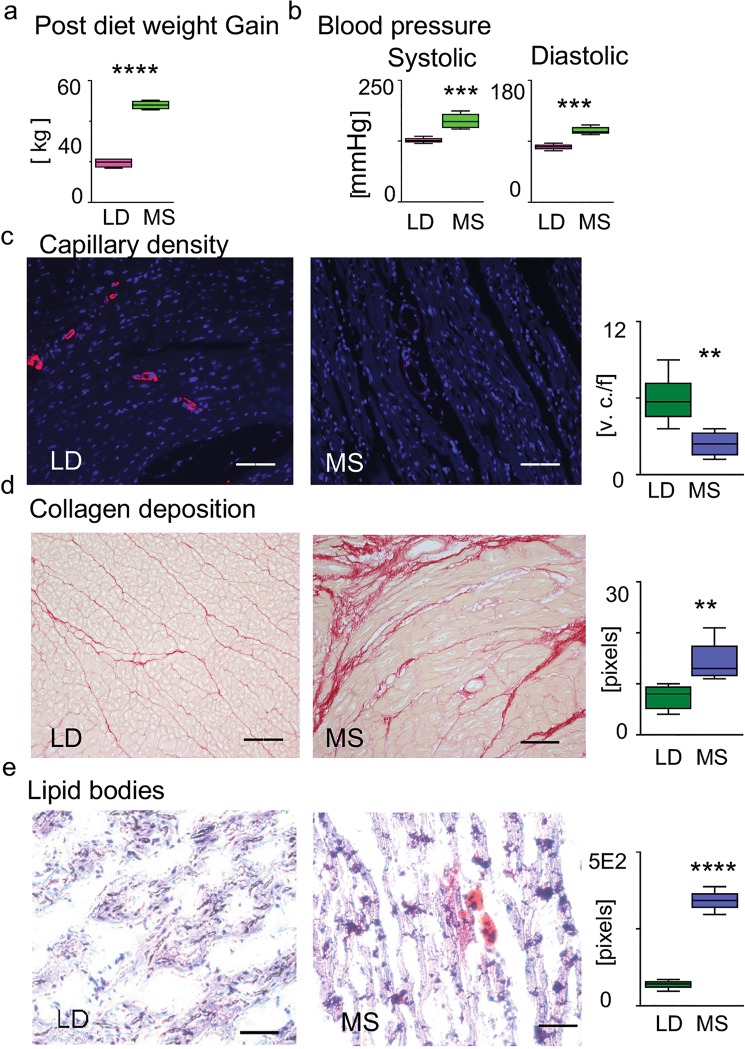
Risk factors related to metabolic syndrome. (**a**) Post-diet weight gain for LD and MS in [kg] (n = 5 for each group, ****p < 0.0001). (**b**) Systolic and diastolic blood pressure [mmHg] in MS and LD pigs (n = 5 for each group, ***p < 0.001). (**c**) Capillary density staining with the endothelial marker CD31- specific antibody (red), DAPI staining for DNA (blue). The bar diagram shows a decrease in capillaries density in MS vs LD (vessel numbers /20xHP field, v.n. / f, each n = 5, **p < 0.01). (**d**) Picrosirius red staining for collagen is measured in pixels (n = 5 for each group, **p < 0.01). (**e**) Oil red O stain of li*p*ids. The intensity of the positive red staining in MS and LD is measured in pixels (n = 5 for each group, **p < 0.01). Error bars indicate ±SEM. Three randomly selected fields were used for the analysis. Scale bars: 20 mm (**c–e**).

The MS pigs showed elevated blood glucose (162 ± 14 mg/dl vs 96 ± 10 mg/dl, *p* < 0.02), triglycerides (1.66 ± 0.5 mmol/L vs 0.66 ± 0.22 mmol/L, *p* < 0.03), plasma LDL (2.64 ± 0.25 vs 0.46 ± 0.12, *p* < 0.01), and total cholesterol (5.8 ± 0.7 mmol/L vs 1.08 ± 0.2 mmol/L, *p* < 0.007I) in MS vs LD. In MS pigs, we further observed increased weight gain (Fig. 1a) and blood pressure (Fig. 1b).

Additionally, our histology stains demonstrated changes at the tissue level that have been associated with CVD pathogenesis^7,8^ including diminished capillary density, as shown by the difference in the immunologically detectable CD31 in the cardiac tissue (Fig. 1c, **p < 0.01); the picro sirius red stain for collagen (Fig. 1d, **p < 0.01); and accumulation of intracellular lipid bodies as shown with Oil Red O staining (Fig. 1e, **p < 0.01).

Together, the phenotype observed in pigs on the high-fat, high-calorie diet meets all five metabolic syndrome diagnostic criteria in humans: obesity, elevated fasting blood sugar, elevated triglycerides and LDL, and increased blood pressure. The high-fat diet, introduced to In meet the clinical criteria for MS, has demonstrable effects on heart tissue composition in pigs: specifically, increased fat, fibrous tissue, and diminished vascularity, suggesting that high-fat diet leads to histopathological changes at the cellular and molecular levels.

### Metabolomic analyses, together with unsupervised learning approaches, uncover diet-related alterations in the abundance of several cardiac polar metabolites

**Figure 2:**
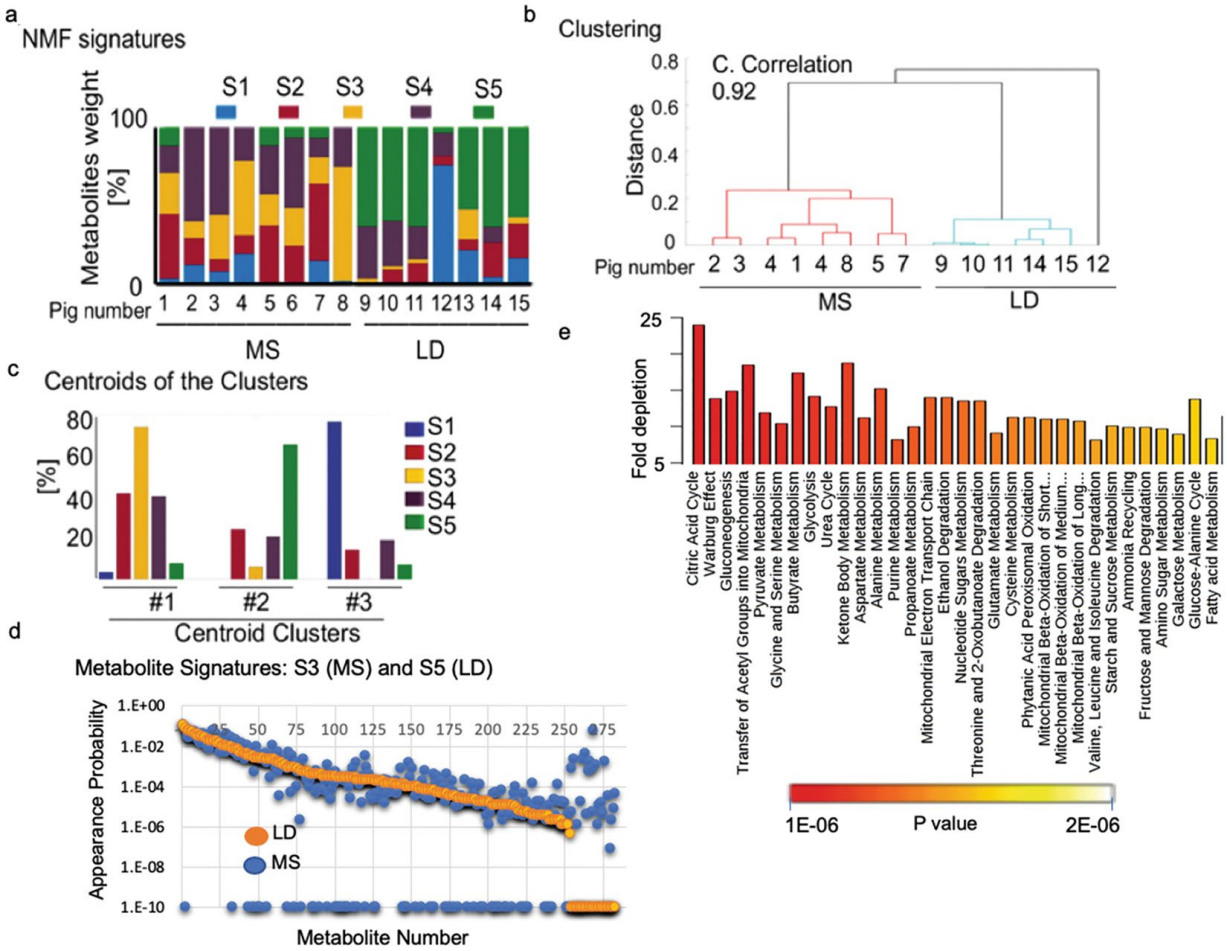
LC/MS-MS and UML identified key metabolic changes in the cardiac response to diet. Acetonitrile and methanol extracts from left ventricular cardiac tissues were analyzed for the presence of polar metabolites by LC/MS-MS. Two hundred eighty-seven polar metabolites were identified and measured in eight individual MS pig and seven lean diet LD controls (Table S1; supplementary). (**a**) UML identifies five signatures (S1, S2, S3, S4, S5 in different colors) of metabolic processes from the mass spec-derived 15-pig data sets. (**b**) UML-based hierarchical clustering of the metabolomics database of the 15 pigs (–cophenetic correlation coefficient 0.92, c. correlation). (**c**) The clusters centroids (%). (**d**) Appearance probability of individual metabolites in signature S3 (blue dots) and S5 (orange). (**e**) Over-represented pathways in signature S5 (LD) vs. S3 (MS). The colored bar shows the *p* values.

To create metabolite signatures from MS and LD heart, we first applied targeted LC/MS-MS for polar metabolites, quantifying the relative abundance of 283 metabolites in each cardiac sample^9^. Tissues were isolated from eight MS and seven LD animals and analyzed by mass spec within a single experiment to avoid potential batch effects. As the variables (283 metabolites) are significantly higher than the replicates (15 pigs), we applied unsupervised machine learning (UML) to compare differences in profiles of metabolites in MS vs. LD^10–12^. UML recognized five distinct profiles (signatures S1, S2, S3, S4, and S5), each with different proportions of the 283 assayed metabolites. Differential weighting of each profile accounts for variations between samples (Fig. 2a). Subsequent clustering of the weights of the metabolite profiles results in two clear groups of samples, one group containing eight MS pigs and another group with the seven LD pigs (Fig. 2b). The centroids of the identified clusters demonstrate the relative contribution of the individual metabolic signatures in the corresponding clusters (Fig. 2c). UML identified S5 as the primary metabolic signature in the LD pigs, while S3 is prevailing in MS. The overrepresented metabolites in S5 are shown in Table S1 (supplementary). The difference between S3 and S5 is further demonstrated by the probability of individual metabolites to appear in the signatures (Fig. 2d).

CAC (*p* = 4e-5), together with glucose and pyruvate (p = 3.1e-4) metabolism predominate in the LD related S5. CAC and pyruvate are, however, significantly diminished in the MS corresponding signature S3 (Fig. 2e). Processes that are involved in the metabolism of butyrate (*p* = 1.1e-4) and the Warburg effect (*p* = 3.3e-5) are diminished in S3 as well. Conversely, processes that are related to glutamate-gamma-aminobutyric acid (GABA) biosynthesis (*p* = 4e-5) and the urea cycle (*p* = 0.004) are overrepresented in S3.

### LD and MS profiles show a distinct metabolite distribution

**Figure 3:**
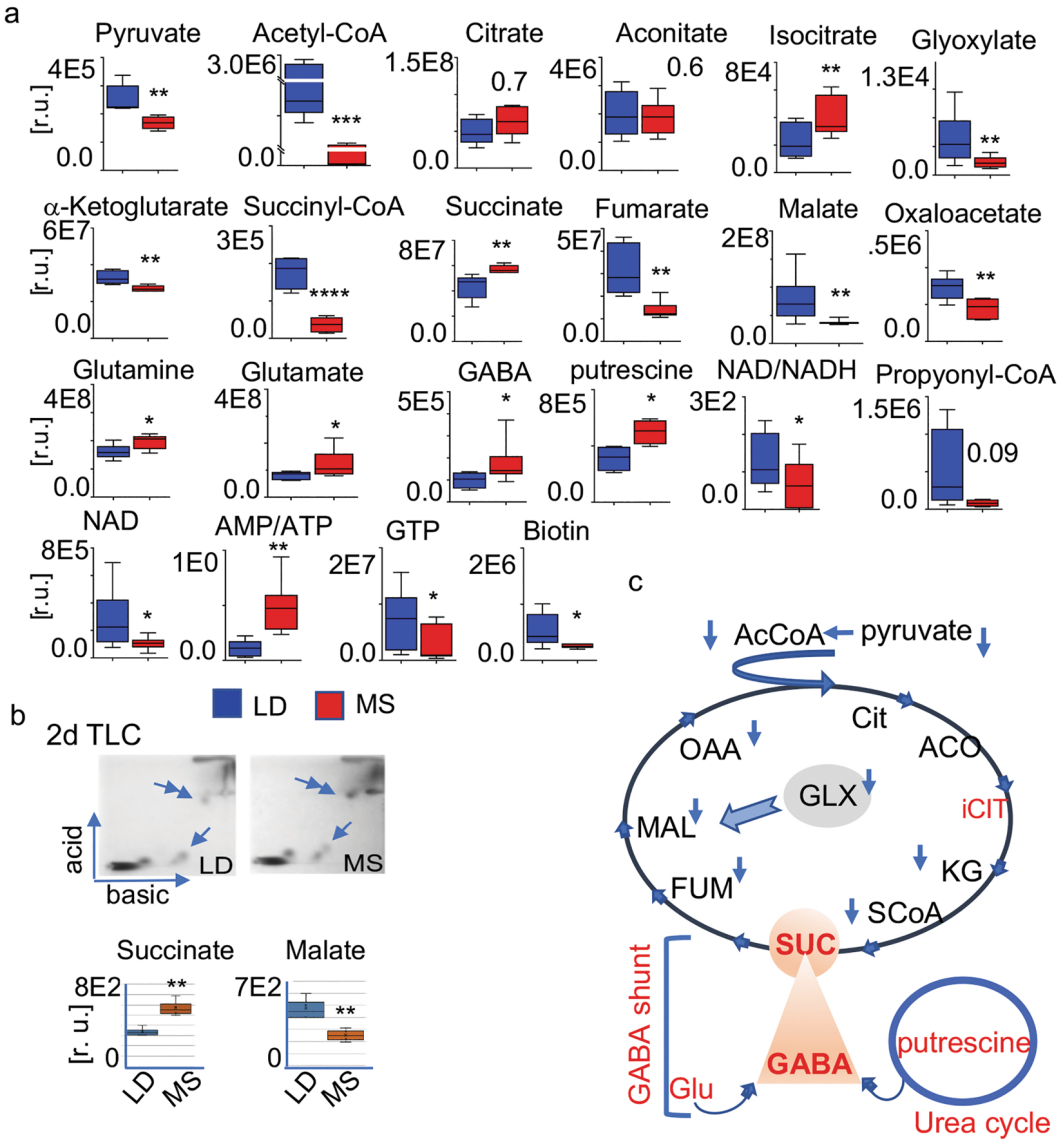
CAC metabolites in MS and LD. CAC related intermediate metabolites in the MS and LD heart are identified and quantitatively compared by LC/MS-MS. Data represents 7 LD and 8 MS pig. (**a**) Content of pyruvate, acetyl CoA, and CAC intermediate metabolites: citrate, aconitate, isocitrate, glyoxylate, a-ketoglutarate (a-KG), succinyl-CoA (SCoA), succinate, ɣ-aminobutyric acid (GABA), glutamate, glutamine, fumarate, malate, oxaloacetate in relative units. The individual metabolites are shown at the top of the diagrams. Values are means ± SD; *p < 0.05, **p < 0.01, ***p < 0.001; LD n = 7, MS n = 8. (**b**) 2d TLC separation of succinate and malate in LD and MS tissue: the single arrow indicates the position of malate; double arrow indicates succinate. The spot intensity (pixels) of four independent extracts, each from 10 mg of tissue (n = 4 and 4, **p < 0.01) is shown with the bar diagram. Error bars indicate ±SEM. (**c**) Diagram representing the alterations in the intermediate CAC metabolites in the MS heart together with the CAC connected glutamate-GABA and glyoxylate pathway: acetyl CoA (AcCoA) and the CAC intermediate metabolites citrate (Cit), aconitate (ACC), isocitrate (iCIT), glyoxylate (GLX), a-ketoglutarate (KG), succinyl-CoA (SCoA), succinate (SUC), ɣ-aminobutyric acid (GABA), glutamate (Glu), fumarate (FUM), malate (MAL), and oxaloacetate (OAA). The blue arrows denote diminished metabolites in MS vs. LD; red - high in MS.

The S3 and S5 profiles likely differ in the content of numerous metabolites related to both CAC and glycolysis (Fig. 3). Therefore, we next compared metabolite abundance in the LD-related S5 and the MS-related S3 using the quantitative mass spec data. The quantitative mass spec results (Fig. 3a) showed a decrease in the CAC entry metabolites acetyl CoA (*p* < 0.001) and pyruvate (*p* < 0.01) in MS. The decreases in the CAC intermediates α-ketoglutarate (*p* < 0.01), succinyl-CoA (*p* < 0.0001), fumarate (*p* < 0.01), malate (*p* < 0.01), and oxaloacetate (*p* < 0.01) are all statistically significant. Glyoxylate (GLY), an intermediate in an alternative CAC pathway was diminished in MS (*p* < 0.01) as well.

Contrariwise, succinate (*p* < 0.01), citrate (*p* < 0.01), and isocitrate (*p* < 0.01) were strikingly elevated in MS. Additionally, glutamine (*p* < 0.05), glutamate (*p* < 0.05), and gamma-aminobutyric acid (GABA, *p* < 0.05) as well as putrescine (*p* < 0.01) from the urea cycle were all increased in MS.

Moreover, while the AMP/ATP ratio is elevated in MS, the NADH content (*p* < 0.01), as well as the NAD + /NADH value (*p* < 0.01) are diminished.

Two-dimensional thin-layer chromatography (2dTLC) was applied to validate observations (Fig. 3b). The semiquantitative 2dTLC of MS (n = 4) and LD (n = 4) samples supported the mass spec data by consistently showing more than a threefold increase in succinate (*p* < 0.01) and significantly less malate (*p* < 0.02) vs LD. Changes in metabolites between the two groups are graphically summarized in Fig. 3c.

Altogether, the comparison of S3 and S5 signatures reveals an imbalance in CAC intermediates in MS. Specifically, we observed significant decreases in the availability of pyruvate and acetyl-CoA at the entry of the CAC, as well as decreases in several downstream CAC intermediates, including alpha-ketoglutarate, malate, and oxaloacetate. The critical CAC cofactor NADH and the NAD + /NADH ratio are altered as well. The disproportionally large accumulation of succinate, the fifth intermediate in CAC, in comparison to succinyl-CoA, fumarate, and malate, coincides with an increase of GABA; increases in the GABA cycle intermediates glutamine and glutamate; as well as elevated putrescine from the urea cycle in MS. Moreover, the considerable accumulation of citrate and isocitrate coincides with a decrease of acetyl-CoA.

### CAC, GABA cycle, and glyoxylate shunt enzymes respond to MS at the transcriptional and protein level

**Figure 4:**
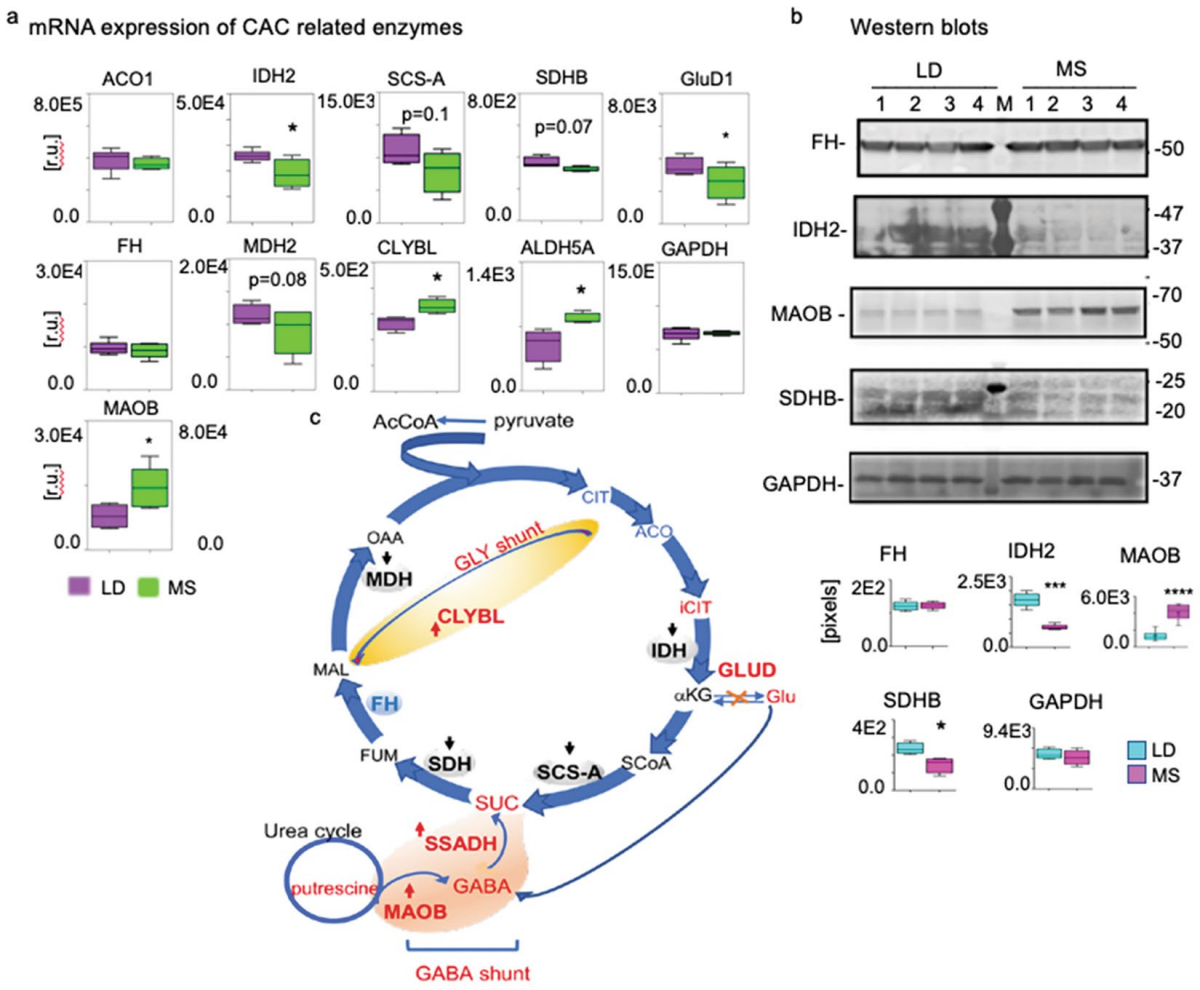
CAC enzymes’ mRNA in MS and LD. (**a**) RNA-seq was applied to identify and compare mRNAs contents of CAC-involved metabolic enzymes. Cardiac mRNA libraries from LD (n = 4) pig and MS (n = 4) pig were compared for relative content of cytosolic aconitase 1 (*ACO1*); isocitrate dehydrogenase from mitochondria (*IDH2)*; succinyl-CoA synthetase beta-a chain (*SCS-A*); succinate dehydrogenase (*SDHB*); fumarase (*FH*); malate dehydrogenase 1 (*MDH1*); citrate lyase beta-like (*CLYBL*); Glutamate-GABA shunt related mitochondrial succinic semialdehyde dehydrogenase (*ALDH5A1*); and glutamate-α-ketoglutarate related glutamate dehydrogenase 1 (*GluD1*). *GAPDH* mRNA content was used as an internal control for equal loading. Values are means ± SEM; *p < 0.05, **p < 0.01; green (MS), purple (LD). (**b**) Western blot with cardiac tissue protein lysates (50 μg/lane) and protein-specific antibodies against FH, IDH2, SDHB, GluD1, and GAPDH were used to verify the RNA-seq data in four MS and four LD pigs as shown above the plots. On the right -the molecular weight migration markers; left - antibody specific signals that are semi quantitatively (pixels) evaluated and presented with the bar diagrams. We represent that data as means ± SEM; ***p < 0.001. The individual membranes that are probed for antibody-protein recognition are shown in Fig. S1, together with the Ponceau S stains that verify equal protein loading and transfer. (**c**) Illustration of the alternations in CAC pathway enzymes in MS vs. LD: blue, enzymes that are down-regulated in MS; red, upregulated in MS; pink, the glutamate-GABA shunt; yellow, the GLY shunt.

To determine the mechanisms of cardiac CAC dysregulation and MS adaptation, we determined and compared at the mRNA levels the expression of enzymes that drive the traditional CAC, GABA cycle, and the glyoxylate shunt (Fig. 4a). RNA-seq data from four of the MS and four of the LD pigs demonstrated diminished levels of several CAC enzymes in MS: isocitrate dehydrogenase (*IDH2*, *p* < 0.05); glutamate dehydrogenase, mitochondrial (*GluD1, p* < 0.05); subunit B of the mitochondrial succinate dehydrogenase (*SDHB*, n = 4, *p* = 0.07), and the mitochondrial malate dehydrogenase (*MDH2*, MS n = 4, *p* = 0.08). We also observed a trend of decreased expression of the mitochondrial succinyl-CoA synthetase (*SCS-A*, MS n = 4, *p* = 0.1) and the cytosolic aconitase (*ACO1*, MS n = 4, *p* = 0.5) in MS vs. LD. Conversely, in the GABA-glutamate-glutamine cycle, mitochondrial succinic semialdehyde dehydrogenase (*ALDH5A1*) is significantly increased in MS vs. control (n = 4, *p* < 0.05). Furthermore, the monoamine oxidase B (*MAOB*), which is known to use putrescin for GABA production increases in MS as well^13^. Additionally, the mitochondrial citrate lyase beta-like (*CLYBL*) that is involved in the glyoxylate shunt, and could convert glyoxylate to malate, is also increased in MS (n = 4, *p* < 0.01)^14^.

Although we noted significantly less fumarate in MS vs. LD, expression of fumarase (FH) did not change in response to diet. Additionally, expression levels of neither glyceraldehyde-3-phosphate dehydrogenase (*GAPDH*) nor the mitochondrial *ACO2* variant showed diet-related mRNA variations in content and served as internal controls for the observations noted above.

Western blots with total cardiac tissue lysates (50 µg protein) from four MS and four LD pig and specific antibodies against FH, SDHB, IDH2, MAOB, and GAPDH supported the RNA-seq observations related to the expression of the corresponding genes (Fig. 4b). Fumarase and GAPDH protein levels broadly reflected the mRNA levels without significant diet-related alterations. The SDHB (*p < 0.05) and IDH2 (****p < 0.0001) protein contents are diminished in MS, reflecting their mRNA levels. Conversely, analogous to the mRNA level, there is significantly more MAOB (***p < 0.001) protein in MS vs. LD. The membranes with the transferred proteins were stained with ponceau S to control for equal transfer and protein loading (Fig. S1; supplementary). Gene expression results are summarized in Fig. 4c.

Combined, these observations support the hypothesis that MS alters the flux of multiple CAC metabolites by changing the expression of genes encoding traditional CAC enzymes along with enzymes that are involved in the *GABA* and glyoxylate shunts.

### Fumarase O-GlcNAcylation coincides with altered enzymatic activity in MS

**Figure 5:**
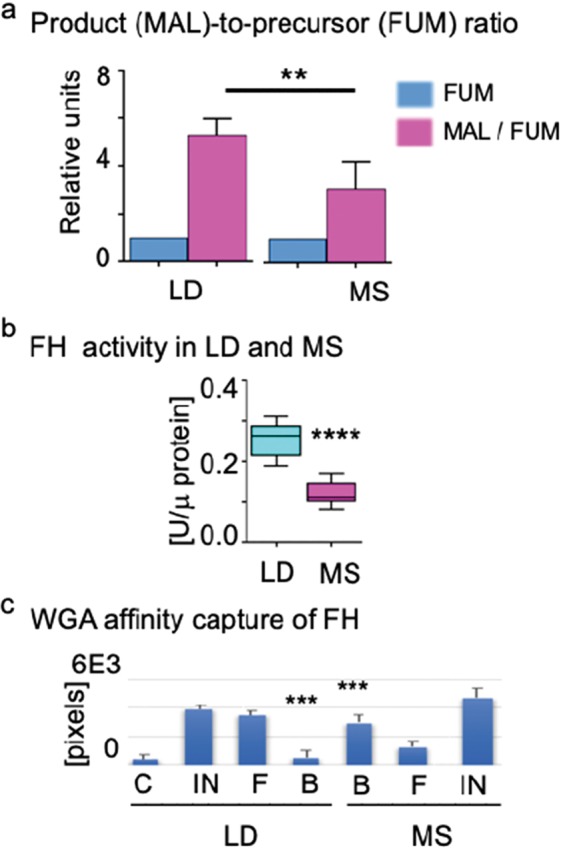
Fumarase O-GlcNAcylation and activity response to MS and LD. (**a**) The bar diagram shows the precursor fumarate (FUM) to malate (MAL) product ratios in LD and MS. The FUM and MAL content is determined based on the LC/MS-MS data in LD (n = 8) and MS pig (n = 7). The FUM content is taken as a base (100%) to calculate the MAL content in a FUM: MAL analysis (454.8 ± 67.09%) in LD and (159.5 ± 27.81%) in MS. The product data are presented as means ± SEM; **p < 0.01, MS (n = 8), LD (n = 7): the precursor and product identities are shown at the bottom of the diagram. (**b**) FH activity was measured in total tissue lysates (10 μg protein/reaction LD n = 4, MS n = 4) at 37 °C. All activity measurements were conducted in duplicate at 37 ^o^C for 20 min, and data are presented as means ± SD; ****p = 0.0001 in relative units/µg protein (U/ µg protein). (**c**) The diagram summarizes the western blot data with the WGA affinity capture of O-GlcNAcylated FH in total protein lysates from MS (n = 4) and LD (n = 4): specific immunological reaction (pixel density) for FH in the input fractions -IN, flow through- F, WGA bound – B, bound to control beads without WGA- C. As a base for comparison is used the FH-positive reaction in the input fraction (100%). Data are represented as means ± SD; ***p < 0.001.

Given the apparent absence of changes in FH protein and mRNA abundance in MS vs LD, we investigated the possibility that posttranslational modification may lead to disruption of FH function and activity, and thus corresponding disturbances in fumarate and malate fluxes in MS. This notion is supported by the precursor-to-product proportion distinction in MS vs. LD (Fig. 5a). Based on the mass spec determined fumarate and malate quantities we determined that their ratio is lower in MS vs. LD (MS, n = 8, 1:2.82 ± 0.14, *p* < 0.01; LD, n = 7, 1:3.56 ± 0.13, *p* < 0.01) by fumarate as 100%.

Next, we used total tissue lysates to measure and compare the FH activity in hydration of fumarate to malate reaction (Fig. 5b). In reactions assembled with 10 μg protein/reaction, at 37 °C FH was found to be significantly less active in MS (n = 4, ****p < 0.0001) vs LD (n = 4).

As FH activity is dependent on enzyme oligomerization^15^, posttranslational modifications such as O-GlcNAcylation could alter the conformation of the enzyme and/or prevent oligomerization of enzyme monomers, thereby diminishing its activity. Previously, we and others have observed that in hyperglycemic conditions, the abundance of O-GlcNAcylated proteins is correspondingly greater than in normoglycemic conditions^16–20^. Because a change in FH activity could explain the significant accumulation of succinate which we registered in the MS heart, we tested the hypothesis that in MS tissue whole-cell lysates, the O-GlcNAcylation state of FH protein will be altered. Wheat germ agglutinin (WGA) affinity binding of O-GlcNAcylated proteins in MS and LD lysates (100 mg) and subsequent western blot (0.05 mg/lane) with FH -specific antibodies revealed significant raise of O-GlcNAcylated FH variant in MS (n = 4, ***p < 0.001) vs. LD (n = 4) (Fig. 5c, lanes B). The finding is consistent through separate studies with the lysates of four MS and four LD pigs. The O-GlcNAcylated FH in the MS and LD fractions was quantified based on the optical density of the immunostaining reactions in the western blots.

These outcomes, considered together, allude to MS-specific FH O-GlcNAcylation that favors a derangement of fumarate and malate levels in MS.

## Discussion

Despite the considerable investigations into physiological, epigenetic, and signaling pathway changes in MS, specific disturbances characterizing the cardiac metabolic state remain unclear. Because of MS’s multifaced nature, single molecular markers and processes may not adequately comprehend its impact on the heart. Essential mechanisms that drive the development of MS remain to be established. To date, we developed the most comprehensive platform studying the cardiac response to MS. Our application of UML to 283 metabolites from MS and LD cardiac pig tissues differs from the previously used pattern-recognition approaches. It allows us the identification of yet undescribed metabolic connections. It licenses us the distinctive confirmation of yet undescribed metabolic affiliations. By incorporating mass spec-based comparative metabolomics, transcriptomics, and functional analyses together with UML, in this study, we discovered unknown pathway links and correlations on numerous biomarkers across all five human MS-associated factors: obesity, elevated fasting blood sugar, triglyceride, LDL level, and blood pressure. Specifically, our UML-based clustering of polar metabolites revealed unforeseen, contrasting changes in CAC-related metabolites, concurrent declines in glycolysis, and evidence of CAC replenishment by GABA-glutamate, GABA-putrescine, and glyoxylate shunting.

MS drives alterations in the CAC, as evidenced by the concurrent decline in pyruvate, the entry point of the CAC, and its breakdown product, acetyl-CoA. Others have previously reported a similar drop in pyruvate and acetyl-CoA accumulation in infarcted hearts of rodents^21^. Currently, the etiology of diminished cardiac glycolysis and acetyl-CoA availability in MS is not well understood. The elevated circulating triglycerides and fatty acids that we registered in MS could overwhelm mitochondrial capacity for fatty acid oxidation, subsequently leading to diminished acetyl-CoA production, though further investigation is warranted.

Interestingly, despite the depletion of many CAC intermediates in MS, succinate, the fifth intermediate in the cycle, is more abundant in MS than in control lean-diet heart. This observation raises two questions: what are the sources of succinate in MS, and what role does succinate accumulation play in MS? The putrescine-GABA and glutamate-GABA cycle may supply succinate in MS; this is supported by the increased GABA abundance and its precursors’ putrescine, glutamine, and glutamate that we observed in MS, as GABA is an established succinate precursor. This cycle may provide recourse for the CAC to progress in MS, though further data are needed.

Propionyl-CoA is in like way rich in MS and could give an additional source of succinyl-CoA and its downstream product, succinate. However, given the diminished levels of succinyl-CoA together with biotin, which is the essential cofactor for propionyl-CoA carboxylase, this is unlikely^22,23^. Together, our data suggest that the putrescine-GABA and glutamate-GABA cycles are the most likely candidates for succinate production in MS.

Until recently, succinate had been functionally considered only as a CAC intermediate. Recent data, however, suggest it may have additional, unexpected signaling functions^24^. As the continuous administration of succinate produces a dose-dependent increase in mean arterial pressure, the observed systolic blood pressures in our MS model could be linked to succinate accumulation^23,24^. Interestingly, metoprolol succinate has shown considerable therapeutic promise in reducing mortality related to heart failure, and it is more effective than other metoprolol formulations. Though succinate is administered as salt to increase the serum half-life of metoprolol, the role of succinate *per se* may be an overlooked benefit in this widely-employed therapy^25^. Given the vital role of succinate in the heart and the dysregulation we report in MS, it has potential as a therapeutic target. Despite increased succinate, levels of fumarate, the following intermediate in the CAC, are diminished in MS. This decrease may be driven principally by the reduced expression of succinate dehydrogenase in MS. As previously reported, fumarate functions not only as a malate precursor in the CAC but also could preserve cellular redox potential, improve calcium homeostasis, diminish free radicals and toxic acyl-CoA derivatives deposition^26^. The low fumarate also correlates with the diminished expression of the hypoxia-inducible factor HIF1A in MS (Fig. S2; supplementary) and altered cardiac angiogenesis in MS as previously reported^27^. Not only is fumarate low in MS, but the possible means of its replenishment through phenylalanine/tyrosine, which is abundant in MS, likely does not occur. This is evidenced by the low levels of phenylalanine hydroxylase and fumarylacetoacetase enzymes in MS (Fig. S2; supplementary).

Likely, the fumarate level is tightly controlled not only at the level of succinate dehydrogenase expression but also at the level of fumarase activity (to convert fumarate to malate). Our observation that O-GlcNAcylated inactive fumarase is relatively abundant in MS may explain the diminished fumarate – malate balance. The multimeric fumarase is a highly potent enzyme that requires no co-substrates or co-enzymes to function^28,29^. The bulky O-GlcNAcyl moiety, however, may alter fumarase oligomerization and function^30^. Despite its centrality, the reaction part of fumarase isn’t inside and out of understanding. As fumarate accumulation could trigger both adaptive and maladaptive responses, appropriately balanced fumarate supplementation is a future avenue in developing strategies to prevent the heart from the negative consequences in MS.

Malate likewise participates not only in CAC but also in other metabolic pathways and may have a protective effect against cardiac ischemic injury^31^. Its balance is likely to be controlled not only by fumarase substrate availability and activity but also by external anaplerotic replenishment. The conversion of pyruvate to oxaloacetate by pyruvate carboxylase (and the subsequent reduction of OAA to malate), however, is less likely to be efficient in MS, as levels of both the enzyme’s substrate (pyruvate) and obligate co-factor biotin are low. Conversely, increased mitochondrial citramalyl-CoA lyase (*CLYBL*) expression and the presence of cardiac glyoxylate may replenish malate in MS. It is commonly acknowledged that the glyoxylate cycle is missing in mammals and exists just in plants and microorganisms. However, its function as an alternate CAC cycle that bypasses the oxidative part of the CAC to replenish malate has been reported to occur in the liver^32^. Glyoxylate in its metabolic network presents a new cardiac paradigm for control of malate flux into the CAC in MS.

Despite decreased acetyl-CoA and oxaloacetate, levels of citrate and isocitrate, the following intermediates in the CAC, are significantly more abundant in MS than in control lean diet-heart. The high fatty acids level in MS may likely contribute to the cardiac citrate accumulation, as previously reported occurring in rat hearts after reperfusion with fatty acids^33^. Citrate is a key intermediate in CAC and fatty acid synthesis and could significantly contribute to the diminished glycolysis in MS^34,35^. Moreover, it could also be a factor in the collateral dysregulation that we registered in the MS heart^36^. A superior comprehension of components managing heart citrate accumulation and discharge may recommend medication or diet in MS patients with CVD.

Low metabolite abundance may reflect either a higher rate of consumption or a lower rate of formation in MS, necessitating additional data to suggest which process is occurring. Our RNA-seq data support the latter conclusion, at least in part, as the mRNAs coding for multiple enzymes necessary for the production of CAC intermediates is diminished in MS. Additionally, as the NAD + /NADH balance regulates the CAC, the lower NAD + /NADH ratio that we observed in MS likely impairs CAC dehydrogenases, except for succinate dehydrogenase although it should be noted that expression of *SDHB* was considerably reduced in our protein study. Dysregulation of CAC enzymes in MS may further drive CVD pathogenesis; indeed, aberrant CAC enzyme levels and activity have previously been described as harming the heart^37,38^. Disturbance of CAC was demonstrated to precede and to be responsible for mechanical failure of the left ventricle^37^. Isocitrate dehydrogenase 2 is the rate-limiting enzyme of the CAC and is also diminished in MS. In particular, decreased expression of IDH2 induces contractile dysfunction in the heart, and it correlates with impaired oxidative decarboxylation of α-ketoglutarate, thereby redirecting CAC intermediates^39^. The lower expression level of *IHD2* that we observe in MS could contribute to the increased cardiac hypertrophy in MS animals^40^.

We observe considerable alterations in the MS heart, suggesting that MS builds the peril of CVD yet, basically changing the cardiac energy balance.

Although our steady-state metabolomic and transcriptomic analyses are snapshots supporting our predictions, any reliable conclusions related to perturbed metabolic fluxes are unavoidably limited. The relatively short time frame of the high-fat diet may limit the long-term effects of MS in this early metabolic syndrome model. Furthermore, the metabolic response may be gender- and age-specific, and we investigated only intact male animals. However, given the relative paucity of data on the development of MS in young animals, especially early in the development of MS, this may also be beneficial to our understanding of MS pathogenesis.

Overall, we report several novel findings: (i) unsupervised ML of 283 polar metabolites from MS and LD heart reveals a decline in CAC and glycolysis in MS. (ii) Specifically, there is diminished availability of metabolites that enter the CAC: pyruvate and acetyl CoA. Downstream CAC intermediates, including succinyl-CoA, fumarate, malate, and oxaloacetate, are also diminished in abundance. (iii) Accumulation of citrate in comparison to its precursors and its product (α-ketoglutarate) suggests for altered fatty acids synthesis and glycolysis. (iv) Accumulation of succinate in comparison to its precursor, succinyl CoA, and its products (fumarate and malate) suggests an alternative pathway leading to succinate accumulation. (v) Increased putrescine, glutamine, glutamate, and GABA further suggest that this alternative method for succinate repletion occurs through the GABA-putrescine and GABA-glutamate shunts. (vi) We additionally observed changes in glyoxylate accumulation in MS.

Observed changes in metabolite levels correlated well with corresponding alterations at the enzyme level. (vii) Several vital enzymes that drive the traditional CAC (isocitrate dehydrogenase, mitochondrial succinyl-CoA synthetase, subunit B of the mitochondrial succinate dehydrogenase, and malate dehydrogenase) all are transcriptionally down-regulated in MS. (viii) Conversely, we observed increased expression of enzymes from the GABA and glyoxylate cycle pathways that correlate with the accumulation of succinate in MS.

(ix) Additionally, low levels of α-ketoglutarate in MS could be explained by the low levels of the enzyme *GluD1* in MS, which usually replenishes this metabolite. (x) Though we do not observe changes in fumarase at the mRNA or protein level in MS, its post-translational O-GlcNAcylation is increased in MS. We further show that this modification coincides with a diminished conversion of fumarate to malate, providing a mechanistic explanation for malate and fumarate derangement in MS.

Together, these observations allude to a cardiac obligation in early MS to alter the availability of enzymes from the GABA, glyoxylate, and glutamate dehydrogenase anaplerotic pathways, which could explain the replenished pools of metabolic intermediates in CAC. Overall, the present data support a model in which high-fat diet-induced transcriptional and post-translational alternations in several standard CAC enzymes results in a paucity (or in the case of succinate and citrate, overabundance) of their associated metabolic substrates or products in the heart. These alterations could represent adaptive attempts to salvage cardiac function or may drive pathogenesis in MS. Although the response of patients and our pug model to MS is nearly identical^41^, further research will clarify which of the reported cardiac effects are adaptive or pathologic in patients. To this end, our pig-based data may provide a molecular basis for the development of new therapeutic targets, including early interventions for CVD development in the increasingly prevalent MS. Our pig-based platform can discover and investigate potential therapeutic targets for MS-associated CVD within pathways that were previously unknown to be associated with the disease.

## Material and Methods

### Animal model

Male intact Yorkshire pigs (n = 15, four to six weeks old) were provided by the Parsons Research, Amherst, MA. Eight pigs (MS) were given a hypercholesterolemic (2248 kcal/daily) diet: seven pigs (LD) were given regular chow (1824 kcal/daily, Sinclair Research, Columbia, MO) for 12 weeks. In 12 weeks, pigs were anesthetized and physiologic measurements were taken, followed by euthanasia via exsanguination. Tissue from identical cardiac left ventricular regions is immediately frozen in fluid nitrogen. All animal tests and procedures were endorsed by the Animal Care and Use Committee at the Rhode Island Hospital based on the animal care and use regulations that are described in the NIH publication number 5377-3, 1996.

### Serological analyses

Prior animal euthanasia blood samples were drawn from the jugular vein. The serum samples are analyzed at the Rhode Island Hospital, Providence, RI laboratory. All analyses are conducted in agreement with the biosafety regulations at the Rhode Island Hospital, Providence, RI.

### Immunological procedures and WGA affinity chromatography

Frozen or formalin-fixed cardiac tissue sections (12 µm in thickness) of left ventricular territory were stained with the following antibodies: anti-OGA, [EPR7154 (B)], (Abcam); α- Smooth Muscle actin – FITC, (Vector); anti-YY1, ChIP Grade (ab38422) (ABcam); anti-SP1, ChIP Grade (ab13370) (ABcam). WGA affinity chromatography was applied as previously reported^20^. Ponceau S membranes staining is used to validate equal protein loading and transfer. BCA Assay kit (Pierce) is used to measure protein concentration. Periodic Acid-Schiff (PAS) staining (Sigma-Aldrich, procedure 395), lipid Oil Red O (Bio Vision, Catalog # K580-24) and picrosirius red staining kit (Polysciences, Inc) were used per manufacturer’s protocols. All analyses are conducted in agreement with the biosafety regulations at the Rhode Island Hospital, Providence, RI.

### RNA-seq

Fresh left ventricular cardiac tissue was extracted with the RNeasy Mini Kit (Qiagen) per the manufacturer’s protocol. The integrity of RNA of the eight samples (4 MS and 4 LD) is ≥ 9.8 as determined with the RNA 6000 Nano Kit (Agilent Technologies). High-output mode sequencing was performed by GENEWIZ (South Plainfield, NJ) on Illumina HiSeq 2500. The porcine reference genome (USMARCv1.0), the STAR aligner^42^ and HTSeq-count, version 0.5.3p9^43^ were used to map and quantify the reads. The Bioconductor package DESeq was used to perform the differential gene expression analysis.

### 2dTLC

Silica gel G plates with fluorescent indicator (Sigma-Aldrich) were used to perform 2dTLC as previously reported using standard molecules as a migration reference^44^. Spots are identified in UV light based on the migration of standard molecules. The experiments are conducted in agreement with the biosafety regulations at the Rhode Island Hospital, Providence, RI.

### Mass spectroscopy (LC/MS-MS)

Water-soluble metabolites were extracted from 100 mg tissue with 1 ml of ice-cold 80% (v/v) methanol and 0.6 ml acetonitrile. Samples were analyzed by LC/MS-MS^9^. MultiQuant v2.0 software (AB/SCIEX) was used for peak areas integration. LC/MS-MS was run for the 15 individual pig samples (15 independent runs). MetaboAnalyst 4.0 was used to identify known pathways. All analyses are conducted in agreement with the biosafety regulations at the Rhode Island Hospital, Providence, RI.

### Unsupervised machine learning (UML)

Nonnegative matrix factorization based UML was applied to analyze the mass spec data as we previously reported^11^. Hierarchical clustering was performed as in^43,45^. Linux clusters at the Los Alamos National Laboratory were used to run the simulations.

### Statistical analysis

Microsoft Excel and Graphpad Prism7 software were used for data analysis. Differences between 2 datasets were statistically compared with *the Student t*-test (GraphPad Software, Inc, San Diego, CA). We represent the data as means ± SD; *p* < 0.05 was considered to be a statistically significant difference. Immunohistochemical results are presented in pixels as average mean intensity pixels /40XHPF + / − SD or as a fold change vs. LD, analyzing six random fields for each pig. Western blot data are presented as fold MS change vs. LD (pixels, +/− standard error of the mean, SD).

## Supplementary information

Supplementary information.

## Data Availability

The RNA-Seq data accession number PRJNA544355 availability under GEO. The UML predicted metabolites with higher probability in LD vs. MS (P4 = 0) are shown in the Supporting document (Table S1). The 283 polar metabolites in MS and LD are available upon request.
