# Supplementary material for: Metabolomics and the pig model reveal aberrant cardiac energy metabolism in metabolic syndrome
Source: Sci Rep. 2020 Feb 26;10:3483. doi: 10.1038/s41598-020-60387-7 (PMC7044421; doi:10.1038/s41598-020-60387-7)

**Metabolomics and the pig model reveal aberrant myocardial energy metabolism in metabolic syndrome**

**Authors:** Maryam Karimi[1], Victoria Petkova[2], John M Asara[2], Michael J Griffin[3], Frank W Sellke[1], Alan R Bishop[4], Boian S Alexandrov[4], Anny Usheva[1, #]

**Institution and Affiliations:** [1]Division of Cardiothoracic Surgery, Department of Surgery, The Warren Alpert Medical School, Brown University, Providence, RI 02903; [2]Beth Israel Deaconess Medical Center, Harvard Medical School, Boston MA 02115; [3]Sam Houston State University, Proposed College of Osteopathic Medicine, Huntsville, TX 77320 [4]Los Alamos National Laboratory, Los Alamos, NM 87545

**TABLE S1** (related to Fig 2a) Metabolites that are overrepresented in LD vs MS as identified by UML based on the weight of the metabolite in the individual signatures. Probability in LD is higher than in MS (100 means MS, S3=0)

| | | |
|---|---|---|
| glyoxylate | NADH | fructose-1,6-bisphosphate |
| pyruvate | acetyl-CoA | NADPH |
| ribose-phosphate | succinyl-CoA | N-acetyl-glutamine |
| uridine | acetoacetate | Glucose-3-phosphate |
| glucose-1-phosphate | 2-oxobutanoate | UDP |
| biotin | anthranilate | ATP |
| guanosine | glucose-6-phosphate | 3-hydroxybutirate |
| 7-methylguanosine | fructose-6-phosphate | Malonyl-CoA |
| AMP | FAD | glutathione glyoxylate |

**Figure S1 (**related to Fig 4b) (a) Western blot with protein specific antibodies against IDH2, SDHB, FH, MAOB, GAPDH as shown on top of the panels. Each gel received total tissue lysate (50 µg/lane) from four of the LD pigs (lanes 1, 2, 3, 4) and four of the MS pigs (5, 6, 7, 8). The migration position and the molecular mass of the protein that is recognized by the antibody is shown on left. (b) Ponceau staining of the membranes confirmed equal lysate loading and transfer.

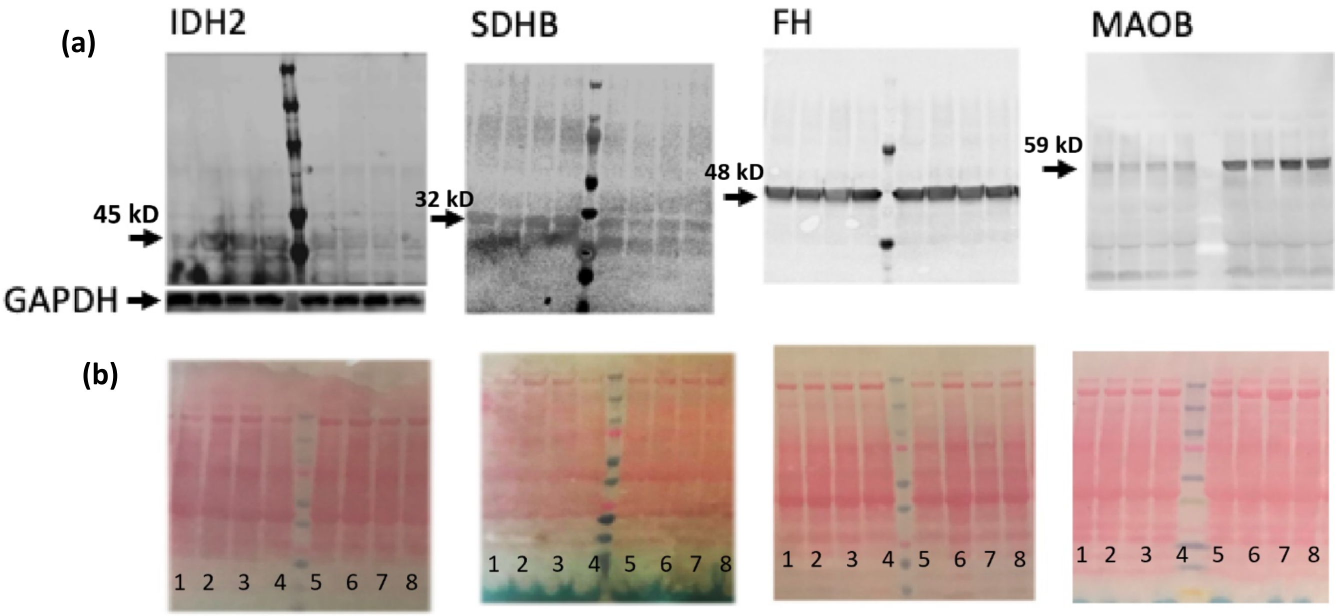


**Figure S2 (**related to Fig 4a) mRNA content of phenylalainine hydroxylase (*PAH*) (n=4, p=0.028) and Fumarylacetoacetase (*FAH*) (n=4, p=0.059)

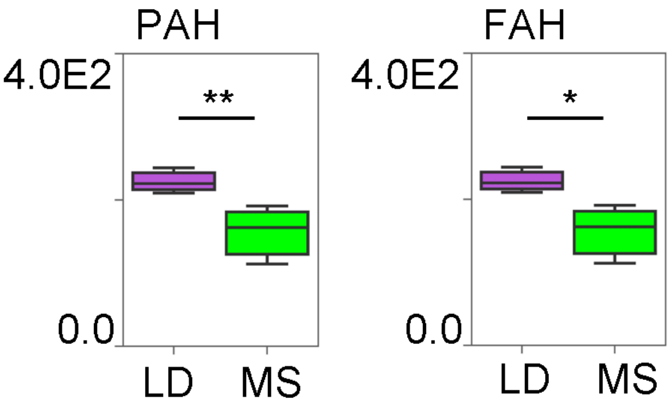

Supplement: Supplementary file 1 — Supplementary information. [file 41598_2020_60387_MOESM1_ESM.pdf]
